# Exploration of parent-reported food allergy symptoms via breastmilk exposures and likelihood to develop tolerance

**DOI:** 10.1186/s13223-021-00606-6

**Published:** 2021-10-09

**Authors:** Abigail Lang, Shrey Patel, Karen Rychlik, Deanna Caruso, Xiaobin Wang, Jacqueline A. Pongracic, Rajesh Kumar

**Affiliations:** 1 Division of Allergy and Immunology, Ann and Robert H. Lurie Children’s Hospital of Chicago, 225 E Chicago Ave, Box 60, Chicago, IL 60613 USA; 2 Department of Pediatrics, Northwestern University Feinberg School of Medicine, Chicago, USA; 3 University of Illinois at Urbana-Champaign, Champaign, USA; 4 Biostatistics Research Core, Stanley Manne Children’s Research Institute, Chicago, USA; 5 Department of Epidemiology, University of North Carolina at Chapel Hill, Chapel Hill, USA; 6 Department of Population, Family and Reproductive Health, Center on the Early Life Origins of Disease, Johns Hopkins University Bloomberg School of Public Health, Baltimore, USA

**Keywords:** Food allergy, Pediatrics, Epidemiology, Breastfeeding, Tolerance

## Abstract

**Background:**

Knowledge is limited about the relationship between clinical reactivity to foods through breastfeeding and long-term food allergy outcomes. We explored parent-perceived symptoms of food allergy via breastfeeding and the association with future tolerance.

**Methods:**

Subjects identified from the Chicago Food Allergy Study (2005–2011) were categorized by parent-reported reactions to maternally ingested foods via breastfeeding (50/898 peanut-allergic, 69/620 egg-allergic, and 153/589 milk-allergic). The primary outcome was tolerance [passed oral food challenge (OFC) or consumption of previously implicated food]. Secondary outcomes included severe reactions (anaphylaxis and/or cardiovascular/respiratory symptoms) and additional concomitant food allergies. Univariate chi-square analyses were performed to assess for association between variables, followed by logistic regression models.

**Results:**

Of the 50 subjects with parent-reported peanut-associated symptoms with breastfeeding, none gained tolerance. There were no significant associations between parent-reported breastfeeding symptoms and development of tolerance for egg and milk (egg: OR 0.46, 95% CI 0.21–1.01, p = 0.053; milk: OR 1.13, 95% CI 0.70–1.81, p = 0.614). All egg-allergic subjects with parent-perceived symptoms while breastfeeding also reported multiple food allergies (n = 69), but milk- and peanut-allergic subjects were not more likely to have multiple allergies (milk: OR 1.89, 95% CI 0.88–4.02, p = 0.10; peanut: OR 2.36, 95% CI 0.72–7.76, p = 0.16). There were no significant associations between parent-reported breastfeeding symptoms and subsequent reaction severity.

**Conclusions:**

A significant proportion of parents perceive symptoms of food allergy attributable to indirect breastfeeding exposures. Our exploratory analysis suggests that infants with parent-perceived clinical reactivity to peanut via breastmilk may be less likely to gain tolerance. Infants with parent-reported reactivity to egg via breastmilk exposure were more likely to report multiple food allergies. Further rigorous prospective studies are needed to clarify the true prevalence of IgE-mediated food allergy symptoms attributable to indirect breastfeeding exposures and the association with development of tolerance.

## Background

Food allergies are common, and little is known about host factors that determine persistence of food allergy versus development of tolerance. The likelihood of developing tolerance varies by the food to which the patient is allergic. Approximately 80% of egg- and milk-allergic patients outgrow their food allergies by adolescence, with up to 50% achieving tolerance by school age [1–3]. Patients with nut allergies are less likely to outgrow their food allergies, with only 20% and 9% of children gaining tolerance to peanut and tree nuts, respectively [4–6]. However, the natural history for each individual food allergy varies significantly between patients, and better predictors of tolerance are needed to personalize and inform patient care.

Prior studies for individual food allergens have demonstrated that patients with higher levels of food-specific IgE (sIgE), larger wheal size on skin prick testing (SPT), and lower rates of decline of sIgE levels are more likely to have persistent food allergies [1, 7]. Additionally, some studies have noted that clinical characteristics such as severity of initial reactions [8, 9], earlier age at presentation [10], and presence of co-morbid atopic conditions [3] may be associated with persistent food allergy. However, other reports have not demonstrated consistent predictors of tolerance across foods for patients allergic to egg, milk, and peanut [11, 12]. There remains a need to investigate additional factors that predict development of tolerance for food-allergic patients.

Research investigating the natural history of developing tolerance to foods in infants who are sensitized and develop symptomatic food allergy through indirect consumption of foods via breastfeeding is even more limited [13, 14]. It is not known how the natural history of food allergy in infants with clinical reactivity to food allergens via indirect exposure through breastmilk differs from children who develop food allergies later in life via direct consumption of the food. Early loss of tolerance in infants reactive to food allergens via breastmilk and the subsequent prognosis of food allergy is not well-studied.

### Objective

The primary objective of this report was to explore the likelihood of developing tolerance among children with parent-reported early IgE-mediated reactions to common food allergens (egg, milk, and peanut) via exposure in breastmilk as compared to those children who developed food allergy symptoms later in life from direct ingestion of the implicated food. We secondarily sought to explore the associations between symptoms of parent-reported food allergy while breastfeeding with the severity of subsequent food allergic reactions via direct consumption of the food and the likelihood of having multiple food allergies.

Based on previous reports that suggest earlier age of presentation of allergy symptoms may correlate with persistence of food allergy [10] and the presumed relative lower amounts of food antigen needed to induce reactions via indirect breastmilk exposure, patients with parent-perceived symptoms of food allergy via breastfeeding exposure may represent a more persistent and more severe phenotype. We hypothesized that patients with parent-perceived symptoms of food allergy via breastfeeding would be less likely to outgrow or become tolerant of the implicated food, more likely to have severe food allergy reactions, and more likely to report multiple food allergies.

## Methods

### Study design

We conducted a retrospective analysis of egg-, milk-, and/or peanut-allergic patients identified from a larger cohort of 1427 children previously enrolled in the Chicago Food Allergy (CFA) Study. The CFA study recruited families in the Chicagoland area between 2005 and 2011. Family inclusion criteria included at least one child 0–21 years of age with food allergy history (categorized as the index subject) and 2 or more family members (including both parents and siblings) who were willing to participate in the study.

Data for each index subject were collected from standardized questionnaire-based interviews of parents. For each food allergy, parents reported the date of onset of symptoms as well as the date of formal diagnosis of each food allergy and the age of the child if or when the food allergy was outgrown [as defined by re-introduction and tolerance of the food, either at home or in a formalized oral food challenge (OFC)]. For each recalled clinical food reaction, we also collected data through standardized questions about types of symptoms, timing of onset of symptoms relative to exposure, treatment and acuity of medical care needed for each reaction, and current status of the food allergy at the time of the survey administration (i.e., resolution with development of tolerance or persistence of food allergy).

Symptomatic food allergy from indirect exposure via breastfeeding was determined by parental report. The primary question to ascertain the presence or absence of breastfeeding symptoms was “[h]as your child ever experienced symptoms to any food that was passed exclusively through breast milk?” as well as a follow-up question to indicate which food was implicated. Another question to support parental report of perceived symptoms of food allergy attributable to breastfeeding included “in a typical week during a period of breastfeeding, how often did you [the mother] eat the […food]?” Additional data about the onset, severity, and types of initial perceived breastfeeding symptoms were not included in the original questionnaire and unable to be collected due to the retrospective design of the study.

Objective results for subjects including corroborative skin prick testing (SPT) and specific IgE (sIgE) levels were obtained at the time of the study visit. Subjects were classified as having persistent food allergy if they met criteria including clinical history of typical IgE-mediated objective symptoms occurring within 2 h of ingestion of the implicated food and sIgE ≥ 95% positive predictive value (based on data from Sampson review in 2004) or positive skin test to the food allergen at the time of the study visit [15]. Data obtained for each index subject included demographic information as well as the presence of additional atopic diseases such as asthma, atopic dermatitis, and allergic rhinitis. Additional information about the index child’s family and household was also collected, including sociodemographic and environmental data. Further information on the methods of the CFA study is described here [16–18].

Subjects were divided into two primary categories based on parent-perceived food allergy symptoms during breastfeeding associated with maternal ingestion of the food. The primary outcome was whether the index child developed tolerance as defined by parental report of either a passed OFC or regular direct consumption of the previously implicated food. Secondary outcomes included severity of subsequent reactions (via direct consumption of the implicated food) and reported history of multiple (more than one) food allergies. Severe food reactions were defined by anaphylaxis including cardiovascular/lower respiratory or laryngeal symptoms based on previously published consensus criteria [19, 20].

### Statistical analysis

Descriptive statistics summarized characteristics of the primary index subject and his/her household and included frequencies and percentages for categorical data and means and standard deviations for continuous data. Univariate chi-square tests and independent t-tests were used to determine associations between predictors of interest and primary and secondary outcomes. Logistic regression models were used to evaluate factors associated with primary outcomes. Adjusted models controlled for subject age and sex, maternal race, household income, and presence of a household pet and included all subjects with non-missing data for all key variables and co-variates. Additional sensitivity analysis models were conducted for the primary outcome (development of tolerance) to adjust for the presence of atopic dermatitis. Models were also constructed for the primary outcome excluding food-allergic subjects without history of breastfeeding. Raw data is presented for outcome variables with zero cells. Complete case analyses were used for all models. All statistical tests were two-sided, and p < 0.05 was considered to be significant. No adjustments were made for multiple tests. Analyses were performed using SAS version 9.4 (SAS Institute Inc, Cary, NC).

## Results

### Patient characteristics

Of the total cohort of 1427 index children enrolled in the CFA study with non-missing data for the primary predictor of interest, 620 reported an egg allergy, 589 reported a milk allergy, and 898 subjects reported a peanut allergy. The majority of subjects with each food allergy were male and white. On average, subjects who outgrew their food allergies were older. The majority of households had an annual income of ≥ $100,000. Most subjects were also sensitized to more than one food and had a history of atopic dermatitis. In univariate analyses, females were more likely to gain tolerance to peanut (p = 0.035). Similarly, egg-allergic and milk-allergic subjects who outgrew their food allergy were more likely to have household incomes ≥ $100,000 and co-morbid allergic rhinitis (p < 0.05). There were no other significant differences in demographic or clinical characteristics between the groups for each food allergen. The percentage of all subjects with parent-reported resolution of food allergy at the time of the survey also varied by allergen with 22% (n = 136) of egg-allergic, 23% of milk-allergic (n = 138), and 3% (n = 25) of peanut-allergic patients reporting development of tolerance. Of those subjects who did not have parent-perceived symptoms of food allergy during breastfeeding, the percentage of subjects who reported resolution of their egg, milk, and peanut allergies were 23% (n = 127), 24% (n = 104), and 3% (n = 25), respectively. Complete demographic data and patient characteristics are summarized in Table 1.

**Table 1:** Summary of the subset of subjects included from the CFA cohort by food allergy

	Egg allergy (n = 620)			Milk allergy (n = 589)			Peanut allergy (n = 898)		
	Current (n = 484)	Outgrown (n = 136)	*p-*value	Current (n = 451)	Outgrown (n = 138)	*p*-value	Current (n = 873)	Outgrown (n = 25)	*p-*value
	N (%)/Mean (SD)	N (%)/Mean (SD)		N (%)/Mean (SD)	N (%)/Mean (SD)		N (%)/Mean (SD)	N (%)/Mean (SD)	
Age (years)	4.9 ± 3.4	7.39 ± 3.6	*< 0.0001*	5.08 ± 3.8	7.62 ± 3.8	*< 0.0001*	6.02 ± 3.7	8.09 ± 4.4	*0.006*
Sex									
Male	336 (80%)	84 (20%)	0.08	290 (75%)	99 (25%)	0.128	593 (98%)	12 (2%)	*0.035*
Female	146 (74%)	52 (26%)		158 (80%)	39 (20%)		278 (96%)	13 (4%)	
Missing	2 (100%)	0 (0%)		3 (100%)	0 (0%)		2 (100%)	0 (0%)	
Maternal race									
White	402 (77%)	119 (23%)	0.262	385 (77%)	115 (23%)	0.525	749 (97%)	22 (3%)	0.567
Other	67 (83%)	14 (17%)		56 (74%)	20 (26%)		104 (98%)	2 (2%)	
Missing	15 (83%)	3 (17%)		10 (77%)	3 (23%)		20 (95%)	1 (5%)	
Household pet									
Yes	202 (75%)	69 (25%)	0.055	208 (76%)	65 (24%)	0.851	385 (97%)	11 (3%)	0.257
No	277 (81%)	65 (19%)		239 (77%)	72 (23%)		479 (97%)	14 (3%)	
Missing	5 (71%)	2 (29%)		4 (80%)	1 (20%)		9 (100%)	0 (0%)	
Household income (annual)									
< $100,000	200 (84%)	39 (16%)	*0.008*	194 (80%)	47 (20%)	*0.049*	307 (97%)	10 (3%)	0.551
≥ $100,000	275 (75%)	94 (25%)		249 (73%)	90 (27%)		552 (98%)	14 (2%)	
Missing	9 (75%)	3 (25%)		8 (89%)	1 (11%)		14 (93%)	1 (7%)	
Parent-reported symptoms while breastfeeding									
Yes	60 (87%)	9 (13%)	0.058	119 (78%)	34 (22%)	0.682	50 (100%)	*0 (0%)*	
No	424 (77%)	127 (23%)		332 (76%)	104 (24%)		823 (97%)	*25 (3%)*	
Multiple food allergies									
Yes	467 (78%)	134 (22%)	0.222	407 (76%)	131 (24%)	0.087	753 (97%)	23 (3%)	0.408
No	17 (89%)	2 (11%)		44 (86%)	7 (14%)		120 (98%)	2 (2%)	
Eczema									
Yes (current or history)	404 (80%)	104 (20%)	0.199	355 (78%)	101 (22%)	0.474	688 (97%)	18 (3%)	0.555
No	73 (74%)	26 (26%)		92 (75%)	31 (25%)		173 (97%)	6 (3%)	
Missing	7 (54%)	6 (46%)		4 (40%)	6 (60%)		12 (92%)	1 (8%)	
Asthma									
Yes (current or history)	197 (75%)	66 (25%)	0.103	171 (74%)	60 (26%)	0.249	386 (97%)	11 (3%)	0.975
No	287 (80%)	70 (20%)		279 (78%)	78 (22%)		485 (97%)	14 (3%)	
Missing	N/A	N/A		1 (100%)	0 (0%)		2 (100%)	0 (0%)	
Allergic rhinitis									
Yes (current or history)	227 (74%)	81 (26%)	*0.01*	212 (72%)	84 (28%)	*0.004*	487 (98%)	11 (2%)	0.237
No	255 (82%)	55 (18%)		239 (82%)	54 (18%)		384 (96%)	14 (4%)	
Missing	2 (100%)	0 (0%)		N/A	N/A		2 (100%)	0 (0%)	

Italicized values statistically significant with *p-*value < 0.05

### Associations of parent-reported breastfeeding symptoms and development of tolerance

A small but significant proportion of subjects had parent-reported food allergy via breastmilk exposure with 11% (n = 69) of egg-allergic, 26% (n = 153) of milk-allergic, and 6% (n = 50) of peanut-allergic subjects endorsing symptoms of clinical reactions via indirect food exposure through breastmilk. For subjects with parent-reported breastfeeding symptoms, 13% (n = 9) of egg-allergic, 22% of milk-allergic (n = 34), and 0% (n = 0) of peanut-allergic patients reported development of tolerance to the previously implicated food at the time of the survey.

The mean food-sIgE (measured at the time of the study visit) was higher overall in individuals who had parent-reported symptoms of food allergy during breastfeeding, but when stratified by persistence of allergy versus development of tolerance, only milk-allergic patients with persistent allergy and parent-reported breastfeeding symptoms had higher sIgE levels when compared to those without parent-reported breastfeeding symptoms (Table 2; p = 0.042).

**Table 2:** Relationship of food-specific IgE to development of parent-perceived symptoms of allergy while breastfeeding

Specific IgE (kU/L)	Persistent allergy			Outgrown allergy		
	No symptoms while BF	Symptoms while BF	*p*-value	No symptoms while BF	Symptoms while BF	*p*-value
	Mean (SD)	Mean (SD)		Mean (SD)	Mean (SD)	
Egg	1.74 ± 1.4	1.92 ± 1.4	*0.415*	0.42 ± 0.5	0.54 ± 0.7	*0.491*
Milk	1.74 ± 1.6	2.15 ± 1.7	*0.042*	0.54 ± 0.6	0.3 ± 0.5	*0.065*
Peanut	2.79 ± 1.8	2.78 ± 1.9	*0.985*	0.21 ± 0.3	–	–

Italicized values statistically significant with *p-*value < 0.05

Of the 50 subjects with parent-reported symptomatic peanut allergy via breastfeeding, none became tolerant of peanut. There were no significant associations of parent-reported breastfeeding symptoms with development of tolerance for egg and milk allergic patients (egg: OR 0.46, 95% CI 0.21–1.01, p = 0.053; milk: OR 1.13, 95% CI 0.70–1.81, p = 0.614) (Table 3). When sensitivity analyses were performed to adjust for the presence or history of atopic dermatitis, there were no significant changes in results. Similarly, adjusted models excluding subjects who had never been breastfed demonstrated no significant differences in results (data not shown).

**Table 3:** Likelihood of development of tolerance in those who reported symptoms while breastfeeding compared to those who developed symptoms from direct ingestion of egg, milk, and peanut

	Egg allergy (n = 620)		OR (95% CI)	*p-*value
	Current (n = 484)	Outgrown (n = 136)		
	n (%)	n (%)		
Parent-reported symptoms while breastfeeding				
Yes	60 (87%)	9 (13%)	0.46 (0.21–1.01)	0.053
No	424 (77%)	127 (23%)	Reference	

	Milk allergy (n = 589)		OR (95% CI)	*p-*value
	Current (n = 451)	Outgrown (n = 138)		
	n (%)	n (%)		
Parent-reported symptoms while breastfeeding				
Yes	119 (78%)	34 (22%)	1.13 (0.70–1.81)	0.614
No	332 (76%)	104 (24%)	Reference	

	Peanut allergy (n = 898)		OR (95% CI)	*p-*value
	Current (n = 873)	Outgrown (n = 25)		
	n (%)	n (%)		
Parent-reported symptoms while breastfeeding				
Yes	50 (100%)	0 (0%)	–	–
No	823 (97%)	25 (3%)		

Adjusted for age, sex, maternal race, presence of household pet, and annual household income

### Secondary analyses

Tables 4 and 5 summarize the associations of parent-reported development of food allergy during breastfeeding for each of the allergens with secondary outcomes of severe food reactions via direct ingestion of the food and presence of multiple food allergies. There were no significant associations between parent-reported symptoms of egg, milk, or peanut allergy via breastmilk exposure and severity of subsequent reactions. There were no mono-allergic subjects in the group with parent-reported allergy symptoms related to egg; all subjects with parent-reported egg allergy symptoms while breastfeeding also reported multiple food allergies (n = 69). Milk- and peanut-allergic patients with parent-reported symptoms of food allergy via breastmilk were not more likely to have multiple food allergies (milk: OR 1.89, 95% CI 0.88–4.02, p = 0.10; peanut: OR 2.36, 95% CI 0.72–7.76, p = 0.16).

**Table 4:** Likelihood of development of severe food allergy reactions in those with parent-reported breastfeeding symptoms compared to those who reported symptoms from direct ingestion of egg, milk, and peanut

	Egg allergy (n = 557)		OR (95% CI)	*p-*value
	Severe reaction (n = 147)	No severe reaction (n = 410)		
	n (%)	n (%)		
Parent-reported symptoms while breastfeeding				
Yes	15 (22%)	52 (78%)	0.79 (0.42–1.46)	0.443
No	132 (27%)	358 (73%)	Reference	

	Milk allergy (n = 532)		OR (95% CI)	*p-*value
	Severe reaction (n = 271)	No severe reaction (n = 261)		
	n (%)	n (%)		
Parent-reported symptoms while breastfeeding				
Yes	64 (44%)	81 (56%)	0.68 (0.45–1.01)	0.053
No	207 (53%)	180 (47%)	Reference	

	Peanut allergy (n = 803)		OR (95% CI)	*p-*value
	Severe reaction (n = 266)	No severe reaction (n = 537)		
	n (%)	n (%)		
Parent-reported symptoms while breastfeeding				
Yes	15 (33%)	30 (67%)	1.00 (0.52–1.93)	0.995
No	251 (33%)	507 (67%)	Reference	

Adjusted for age, sex, maternal race, presence of household pet, and annual household income. Data presented for all subjects with non-missing data for predictor of interest and co-variates

**Table 5:** Likelihood of development of multiple food allergies in those with parent-reported symptoms while breastfeeding compared to those who developed symptoms from direct ingestion of egg, milk, and peanut

	Egg allergy (n = 586)		OR (95% CI)	*p-*value
	Additional food allergies (n = 568)	No additional food allergies (n = 18)		
	n (%)	n (%)		
Parent-reported symptoms while breastfeeding				
Yes	69 (100%)	0 (0%)	–	–
No	499 (97%)	18 (3%)		

	Milk allergy (n = 561)		OR (95% CI)	*p-*value
	Additional food allergies (n = 511)	No additional food allergies (n = 50)		
	n (%)	n (%)		
Parent-reported symptoms while breastfeeding				
Yes	139 (94%)	9 (6%)	1.89 (0.88–4.02)	0.101
No	372 (90%)	41 (10%)	Reference	

	Peanut allergy (n = 855)		OR (95% CI)	*p-*value
	Additional food allergies (n = 736)	No additional food allergies (n = 119)		
	n (%)	n (%)		
Parent-reported symptoms while breastfeeding				
Yes	44 (94%)	3 (6%)	2.36 (0.72–7.76)	0.157
No	692 (86%)	116 (14%)	Reference	

Adjusted for age, sex, maternal race, presence of household pet, and annual household income. Data presented for all subjects with non-missing data for predictor of interest and co-variates

## Discussion

Our study explored the relationship between parent-perceived symptomatic food allergy to egg, milk, and peanut through breastmilk exposure and the subsequent likelihood of gaining tolerance. We hypothesized that infants with parent-reported symptoms of food allergy while breastfeeding would be less likely to outgrow their food allergy, and secondarily, would be more likely to go on to have severe food allergy reactions and multiple food allergies.

As expected, subjects in our cohort with history of egg or milk allergy (with or without parent-reported breastfeeding symptoms) developed tolerance more often than peanut-allergic subjects. Some subjects allergic to egg (11%, n = 69/620), milk (26%, n = 153/589), and/or peanut (6%, n = 50/898) had parent-reported initial reactions to food antigen exposure through breastmilk. Our findings of higher proportions of subjects with parent-reported symptomatic egg and milk allergy during breastfeeding as compared to peanut is consistent with previously published reports [14, 21], which may be in part due to the more frequent consumption of milk and egg in maternal diets as compared to peanut. While there are few estimates of the true prevalence of IgE-mediated allergic reactions attributable to exposure to food antigens expressed in breastmilk (partially due to the difficulty in accurately confirming these diagnoses), there are case reports of anaphylaxis and cutaneous reactions in exclusively breast-fed infants attributable to maternal food antigen consumption [22–24].

In our cohort, all subjects who had parent-reported clinically reactivity to peanut with breastmilk exposure remained allergic to peanut. While not statistically significant, there was also a trend for egg-allergic subjects with parent-reported breastfeeding symptoms to be less likely to become tolerant to egg (OR 0.46, p = 0.052). We did not find the same association for subjects allergic to milk. Our findings did not change when we included sensitivity analyses accounting for the possibility that co-morbid atopic dermatitis might explain the higher rates of food sensitization via cutaneous exposure and the persistence of food allergy in these subjects. We also found no significant differences in our results when we excluded subjects without history of breastfeeding from analysis for the primary outcome of development of tolerance.

Subjects with parent-reported symptoms of egg allergy through breastfeeding were also more likely to report allergies to more than one food. While not statistically significant, subjects with milk and peanut allergy with parent-reported symptoms via breastfeeding also tended to have more than one food allergy. We did not find any associations between parent-reported breastfeeding symptoms and severity of subsequent clinical reactions via direct consumption of the food, and milk-allergic patients with parent-reported breastfeeding symptoms tended to be less likely to have severe reactions (OR 0.68, 95% CI 0.45–1.01, p = 0.053). This may be in part due to inclusion of a higher proportion of younger infants and children in our study as food allergy reactions in these populations tend to have milder symptoms when compared to toddlers and older children [25].

Published guidelines now recommend early introduction of peanut to infants at age 4–6 months to prevent peanut allergy, and it is thought that early introduction of other highly allergenic foods may also help prevent food allergy [26–29]. It has been postulated that earlier introduction to food antigens through breastmilk may also prevent sensitization to common food allergens, although controlled trials investigating this hypothesis have not been performed [26]. Our findings suggest that infants with parent-perceived symptoms during breastfeeding may actually be less likely to develop tolerance. One possible explanation for these findings may be that the amount of food antigen expressed in breastmilk may not be of sufficient quantity to promote development of tolerance. For example, the LEAP study recommended regular consumption of 6–7 g of peanut protein per week [29]. While studies have noted variability in the expression of food antigens in human milk, the amounts of antigen in breastmilk are likely less than what infants would ingest when they directly consume the food [14], and some reports have suggested that the amount of cow’s milk protein present in breastmilk may be below the threshold needed to induce reaction in cow’s milk allergic patients [30]. Additionally, a study in Canada demonstrated that among mothers who consumed peanut while breastfeeding, infants with delayed direct peanut introduction until 12 months of age had higher rates of peanut sensitization as compared to infants with peanut introduction before age 12 months [31]. It is possible that the smaller amounts of antigen contained in human milk may trigger the immune system to shift away from tolerance towards development of a food allergy and a Th2 type response in infants otherwise predisposed to food allergy or atopy without concomitant direct ingestion of the allergen by the infant. Another possible explanation of the differences in persistence of allergies in infants with symptoms during breastfeeding may be changes in the microbiome. Previous studies have demonstrated that the microbiota of infants with food allergy differs from healthy controls; similarly, patients with persistence of food allergy have been shown to have different compositions of the microbial gut flora as compared to those patients who outgrew his or her food allergy [32–34].

We recognize that there are significant limitations to our study. Data about clinical history of food reactions and breastfeeding were collected retrospectively. Recall bias must be considered when interpreting our results as we relied on parent or guardian report of initial diagnosis of food allergy and symptoms of allergic reactions through breastmilk exposures. Additionally, the high percentage of subjects with parent-reported breastfeeding symptoms in our study is likely over-reported. Previous studies have shown that the perceived food allergy reported by parents is higher than the true prevalence in the general population [35], and subjects with severe or persistent food allergy may be more likely to have parents report symptoms attributable to breastfeeding exposures. Specifically, we also did not assess parental levels of anxiety. There may be some differences in how parents perceive the relevance of symptoms with different food ingestions (i.e., egg and milk are more likely to be outgrown and may be perceived as “less severe” than a peanut allergy with a more persistent natural history) leading to differential reporting of parent-perceived breastfeeding symptoms. Details about the timing and types of symptoms as well as severity of reactions attributable to breastmilk exposures were not available in the original retrospective dataset. This likely led to some parental classification of symptoms of worsening of atopic dermatitis, allergic proctocolitis, or other non-IgE mediated symptoms as symptomatic allergy attributable to food exposures through breastmilk which would account for the higher-than-expected percentage of subjects with reported parent-perceived reactions to breastmilk. Although study personnel did review each reported food allergy to determine if reactions met criteria for true IgE-mediated allergy and evaluated objective measures of sensitization at the time of the study visit, we were also limited by the lack of using systematic OFC as a standard means of diagnosing and/or confirming a food allergy or gain of tolerance.

The subjects included in our analysis were recruited from a single metropolitan area, and our cohort consisted mainly of white and middle-class subjects which limits the generalizability of these results. While sensitivity analyses to account for the presence of atopic dermatitis were conducted, we did not adjust for the presence of food allergen in environmental dust in the home. Environmental allergen exposures may play a role in indirect sensitization to foods which may represent a confounding factor in the subset of participants who reported symptoms to food allergens via breastfeeding [36].

Furthermore, our study is limited by selection bias. We noted that rates of outgrowing egg, milk, and peanut allergies in our cohort were much lower than reported in the general food allergy population [1–4], but this may in part be due to the nature of the study design with inclusion of a large number of families who self-selected to participate due to the presence of persistent food allergies. Families of children who have not outgrown their food allergies are more likely to have ongoing involvement in food allergy support groups and willingness to participate in studies. This selection bias is also reflected in the inclusion of many children who were young and more likely to outgrow their food allergies in our study. Previous cohorts have demonstrated that over half of patients who outgrow their egg and milk allergies develop tolerance by 5 to 6 years of age [1–3], and up to 22% of peanut-allergic children may gain tolerance by the age of 4 [6]. Similarly, some patients in the study who outgrew milk and egg allergies may have had multiple food allergies (including more persistent allergies such as peanut) which may have made them more likely to participate in the study. However, there should be no selection bias that would influence participation in the study by presence of food allergy symptoms due to indirect allergen exposure via breastmilk. As such, our finding that no peanut allergic children who developed symptoms via breastmilk developed tolerance is still valid, although admittedly few subjects overall outgrew their peanut allergy in this cohort. Additionally, we found that egg-allergic patients who developed symptoms via breastfeeding also showed a trend, albeit not significant, towards decreased likelihood of tolerance which adds some corroborative evidence.

Our study did not utilize random population-based sampling, and the significant recall bias limitations limit the conclusions that can be drawn from our study. However, studies investigating symptomatic food allergy attributable to breastmilk are rare and difficult to perform with large numbers of participants due to inherent limitations in confirmation of symptomatic food allergy in infants with indirect breastmilk exposures. Our study provides initial data that suggests that subjects with parent-perceived reactions to foods via breastmilk may differ from children who develop food allergy via direct consumption of the implicated food. Our study opens the door for future prospective studies to confirm or refute our findings with confirmation of IgE-mediated food allergy symptoms attributable to breastmilk and longitudinal follow-up.

## Conclusion

This study is the largest investigation specifically assessing the association of parent-reported food-allergy symptoms related to indirect consumption of food allergens via breastfeeding and the subsequent development of tolerance. Our study demonstrates that a large percentage of parents perceive that their children have clinical symptoms attributable to foods via indirect breastmilk exposure. Additional investigation of infants with parent-perceived clinical reactivity to peanut and egg via breastmilk exposure is needed given the possibility that these children may be less likely to outgrow their food allergy or may be more likely to have additional food allergies, respectively. Further studies with large numbers of infants are needed to assess if the association demonstrated in our cohort is present in prospectively recruited food allergic subjects, with additional investigations to see if other genetic or environmental factors modify these results.

## Data Availability

The data that support the findings of this study are available from the corresponding author upon reasonable request.

## Abbreviations

OFC: Oral food challenge
SPT: Skin prick testing
sIgE: Specific IgE
